# Simple Death Risk Models to Predict In-hospital Outcomes in Acute Aortic Dissection in Emergency Department

**DOI:** 10.3389/fmed.2022.890567

**Published:** 2022-05-23

**Authors:** Lingyu Xing, Yannan Zhou, Yi Han, Chen Chen, Zegang Dong, Xinde Zheng, Dongxu Chen, Yao Yu, Fengqing Liao, Shuai Guo, Chenling Yao, Min Tang, Guorong Gu

**Affiliations:** ^1^Department of Emergency Medicine, Zhongshan Hospital, Fudan University, Shanghai, China; ^2^Suzhou Zhi Zhun Medical Technology Co., Ltd., Suzhou, China; ^3^Department of Radiological Medicine, Zhongshan Hospital, Fudan University, Shanghai, China; ^4^CANON Medical Systems (China) Co., Ltd., Shanghai, China

**Keywords:** acute aortic dissection (AAD), in-hospital outcomes, maximum false lumen diameter, site of intimal tear, pericardial effusion, nomogram

## Abstract

**Objective:**

We sought to find a bedside prognosis prediction model based on clinical and image parameters to determine the in-hospital outcomes of acute aortic dissection (AAD) in the emergency department.

**Methods:**

Patients who presented with AAD from January 2010 to December 2019 were retrospectively recruited in our derivation cohort. Then we prospectively collected patients with AAD from January 2020 to December 2021 as the validation cohort. We collected the demographics, medical history, treatment options, and in-hospital outcomes. All enrolled patients underwent computed tomography angiography. The image data were systematically reviewed for anatomic criteria in a retrospective fashion by three professional radiologists. A series of radiological parameters, including the extent of dissection, the site of the intimal tear, entry tear diameter, aortic diameter at each level, maximum false lumen diameter, and presence of pericardial effusion were collected.

**Results:**

Of the 449 patients in the derivation cohort, 345 (76.8%) were male, the mean age was 61 years, and 298 (66.4%) had a history of hypertension. Surgical repair was performed in 327 (72.8%) cases in the derivation cohort, and the overall crude in-hospital mortality of AAD was 10.9%. Multivariate logistic regression analysis showed that predictors of in-hospital mortality in AAD included age, Marfan syndrome, type A aortic dissection, surgical repair, and maximum false lumen diameter. A final prognostic model incorporating these five predictors showed good calibration and discrimination in the derivation and validation cohorts. As for type A aortic dissection, 3-level type A aortic dissection clinical prognosis score (3ADPS) including 5 clinical and image variables scored from −2 to 5 was established: (1) moderate risk of death if 3ADPS is <0; (2) high risk of death if 3ADPS is 1–2; (3) very high risk of death if 3ADPS is more than 3. The area under the receiver operator characteristic curves in the validation cohorts was 0.833 (95% CI, 0.700–0.967).

**Conclusion:**

Age, Marfan syndrome, type A aortic dissection, surgical repair, and maximum false lumen diameter can significantly affect the in-hospital outcomes of AAD. And 3ADPS contributes to the prediction of in-hospital prognosis of type A aortic dissection rapidly and effectively. As multivariable risk prediction tools, the risk models were readily available for emergency doctors to predict in-hospital mortality of patients with AAD in extreme clinical risk.

## Introduction

Acute aortic dissection (AAD), which belongs to a family of acute aortic syndromes including intramural hematoma (IMH), penetrating aortic ulcer (PAU), and thoracic aortic rupture, is a life-threatening clinical condition associated with high morbidity and mortality rates (1, 2). It requires prompt diagnosis and timely interventional therapy to optimize in-hospital and long-term outcomes. The incidence of AAD in Taiwan China is 4.3 cases per 100,000 people per year, similar to that in Europe and America (3, 4). It is three times more common in men than in women, although women present older than men at the onset of presentation and experience worse outcomes (5–7). Systemic hypertension, atherosclerosis, Marfan syndrome (commonly seen in patients aged < 50 years), cocaine use, bicuspid aortic valve, and iatrogenic causes by far are more frequent risk factors in AAD patients. And more patients are treated with interventional procedures timely: open surgery in type A AAD and endovascular therapy in type B AAD (8–12).

With its wide application and rapid accessibility, computed tomography angiography (CTA) has been the preferred diagnostic imaging modality in acute settings (5, 8). Some image features provided crucial diagnostic information, had prognostic value, and were helpful to optimize treatment. Previous studies on type B AAD suggested that the strongest independent predictors of complications including aneurysmal growth and the need for late intervention were an initial false lumen (FL) diameter ≥ 22 mm, a maximal aortic diameter ≥ 40 mm, a patent or partially thrombosed FL, and an initial entry tear (ET) ≥ 10 mm (13, 14). With these changes in care, the in-hospital mortality for type A AAD has decreased significantly from 31 to 22% (5, 6).

Even with the progress in clinical practice, diagnostic imaging, clinician awareness, and treatment strategy, AAD patients still died of time delay, risk transfer (especially type A AAD patients who are initially referred to community hospitals and then transferred to tertiary hospitals with expertise and whole experience), patient refusal (patients with advanced age, critical comorbidity, and those who cannot afford the operations), and the surgical procedure itself in clinical practice (1, 5, 15, 16). Whereas, early surgical repair can decrease crude mortality, there is sparse data on which patients will benefit from such therapy. Moreover, as emergency doctors in such acute settings, it remains a challenge for us to make a rapid and correct prognosis prediction for AAD patients. This study aimed to find a bedside prognosis prediction model based on clinical and image parameters to determine the in-hospital outcomes of AAD in the emergency department (ED).

## Materials and Methods

### Study Population

The acute aortic syndrome (AAS) database of ED of Zhongshan Hospital, Fudan University, was searched for training cohort patients with clinical suspicion of AAD diagnosed between January 2010 and December 2019 retrospectively. In addition, we prospectively enrolled validation cohort patients with clinical suspicion of AAD diagnosed between January 2020 and December 2021. The diagnostic criterion for AD was a classic double-lumen aorta with a visible intimal tear shown by CTA (17). Patients diagnosed as AAD by local hospitals, but in fact, the angiography in our hospital implied IMH only, PAU, or localized AD were excluded. Patients with incomplete image data, circumstantial evidence (e.g., computed tomography pulmonary angiography (CTPA) or echocardiogram showed the presence of AD), or lack of CTA in our hospital were also excluded. Furthermore, patients who visited our hospital after more than 14 days from symptom onset or postoperative follow-up were not included in this study. A flowchart to illustrate this study is shown in Supplementary Figure 1. The Stanford system was used to distinguish the anatomical classification of the affected aorta (18). The study was approved by the Ethics Committee of Zhongshan Hospital, Fudan University (Shanghai, China). The informed consent was obtained from patients or their legal surrogates before enrolment.

### Clinical and Image Parameters

All patients in two cohorts received standard medical treatment in ED, including blood pressure control, heart rate control, and pain relief when necessary (19). We collected the demographics, medical history, treatment options, and in-hospital outcomes. All enrolled patients underwent CTA in our hospital. A series of CT images were collected, including the extent of the dissection, the site of the intimal tear, the ET diameter, the maximal aortic diameter, the maximum false lumen diameter (MFL, the false lumen diameter of the aortic segment where the ratio of true lumen diameter to false lumen diameter is the most minor) (Supplementary Figure 2), and the presence of pericardial effusion. Three professional radiologists analyzed the image data systematically.

### Statistical Analysis

Shapiro–Wilk test was used for testing the normality of all continuous variables. Normally distributed continuous variables were expressed as means ± SD, while abnormally distributed continuous variables were expressed as median (the 25th and 75th quartiles). Categorical variables were presented as frequencies and percentages (%). To determine significant variables between surviving and non-surviving groups in AAD and type A AAD derivation cohort, chi-squared tests were performed for categorical variables, and Wilcoxon rank-sum and one-way ANOVA tests were performed for continuous variables. Logistic regression was performed to develop fast-to-use prognostic models for AAD and type A AAD patients. All analyses were conducted using R (version 3.6.3) and SPSS (version 25). All statistical analyses were two-sided, and the significance level was set to *p* < 0.05.

### Derivation and Validation of a Prognostic Model for AAD

The variables significantly associated with mortality in the AAD derivation cohort were enrolled into the multivariable regression. The AAD prognostic model was constructed based on the significant variables obtained from the multivariable analysis. The likelihood ratio test was used to compare the goodness-of-fit of nested models. The concordance index (C-index) was used to assess the discrimination performance of the nomogram, while the calibration curve was used to analyze the agreement between the nomogram and actual observation. The accuracy of the model was assessed by analyzing the area under the receiver operating characteristic curve (AUC). The prediction model was validated first internally by using 20 repetitions of 10-fold cross-validation within the derivation cohort, and then externally in the validation cohort by using predictions based on the derivation cohort. In the internal validation, discrimination and calibration of the model were assessed *via* cross-validated AUC and Nagelkerke *R* square. In the external validation, model performance was assessed by the same measures used for the primary analysis.

### Derivation and Validation of 3ADPS for Type A AAD

Logistic regression was also performed to screen for risk factors in the type A AAD derivation cohort. Then 3-level type A aortic dissection clinical prognosis score (3ADPS) was constructed based on the significant variables obtained from the multivariable analysis. We assigned points for each variable according to the regression coefficient. The AUC was used to assess the accuracy of 3ADPS.

## Results

### Baseline Characteristics of AAD Patients in the Derivation and Validation Cohorts

A total 939 patients who presented with AAD from January 2010 to December 2021 were collected. After excluding patients with incomplete image data, diagnosed as IMH or PAU, and visiting our hospital after more than 14 days from symptom onset and postoperative follow-up, 449 patients were enrolled in the derivation cohort and 120 in the validation cohort (Supplementary Figure 1).

Of the 449 patients in the derivation cohort, 345 (76.8%) cases were male, and the mean age was 61 years. And 298 (66.4%) cases had a history of hypertension, 13 (2.9%) had a history of MFS, and 77 (17.1%) had a history of aortic aneurysm (AA). Surgical repair was performed in 327 (72.8%) cases in the derivation cohort. And the overall crude in-hospital mortality was 10.9%. In the validation cohort, 93 of 120 (77.5%) were male, and the mean age was 58. Baseline characteristics of the enrolled patients are presented in Table 1.

**Table 1:** Baseline characteristics of AAD patients in the derivation and validation cohorts.

	**Derivation cohort**	**Validation cohort**	***P*-Value**
	**(*n* = 449)**	**(*n* = 120)**	
Age	61 (50, 69)	58 (47, 67)	0.107
Sex, male	345 (76.8%)	93 (77.5%)	0.878
Stanford A	132 (29.4%)	67 (55.8%)	<0.001
Stanford B	317 (70.6%)	53 (44.2%)	<0.001
Hypertension	298 (66.4%)	81 (67.5%)	0.816
Diabetes	35 (7.8%)	9 (7.5%)	0.914
MFS	13 (2.9%)	7 (5.8%)	0.121
History of aortic aneurysm	77 (17.1%)	8 (7.4%)	0.004
Surgical repair	327 (72.8%)	98 (81.7%)	0.048
Open surgery	91 (20.3%)	50 (41.7%)	<0.001
Endovascular therapy	237 (52.8%)	47 (39.2%)	0.008
In-hospital mortality	49 (10.9%)	14 (11.7%)	0.815

It should be noted that the number of patients with type A AAD and patients undergoing surgical repair in the validation cohort were significantly higher than those in the derivation cohort. As we all know, an institutional combination of multidisciplinary expertise in complex surgical repair and established resources and infrastructure are indispensable for the successful estimation and management of AAD, primarily type AAD (20, 21). More patients with type A AAD had been thus transferred to our hospital in recent 2 years, and the operation rate had also increased.

### Comparison Between Surviving and Non-surviving AAD Patients in the Derivation Cohort

The comparison of clinical characteristics and image parameters between surviving and non-surviving groups of AAD patients in the derivation cohort are shown in Supplementary Table 1. There was no significant difference in gender and age between the two groups. Patients with type A AAD and Marfan syndrome (MFS) had a high risk of death (*p* < 0.001), with type A AAD in 99/400 (24.8%) patients of survivors and 33/49 (67.3%) of non-survivors and MFS in 8/400 (2%) patients of survivors and 5/49 (10.2%) of non-survivors. More patients in the surviving group received surgical repair (75.8 vs. 46.9%, *p* < 0.001) and had a longer time window from symptom onset to operation (8 vs. 4 days, *p* < 0.001).

Image parameters showed that patients with dissection involving the aortic sinus, brachiocephalic trunk, left common carotid artery, and left subclavian artery had a worse prognosis (14.3 vs. 3, 28.6 vs. 8, 24.5 vs. 7.5, 22.4 vs. 8.5%, respectively, *p* < 0.002). Patients with an entry tear at the ascending aorta had a high risk of death (34.7 vs. 12.5%, *p* < 0.001). And the ET diameter and MFL diameter in the non-surviving patients were dramatically larger than that in the surviving group (7.1 vs. 3.9 mm, *p* = 0.001; 27.5 vs. 24.5 mm, *p* = 0.019, respectively). Additionally, patients with pericardial effusion were much more in the non-surviving group than in the surviving group (28.6 vs. 10%, *p* < 0.001).

### Comparison Between Surviving and Non-surviving Type A AAD Patients in the Derivation Cohort

The comparison of clinical characteristics and image parameters between surviving and non-surviving groups of type A AAD patients in the derivation cohort are shown in Supplementary Table 2. Undoubtedly, more patients in the surviving group received surgical repair (79 vs. 37.5%, *p* < 0.001), and most of the patients in the surviving group received open surgery (71 vs. 37.5%, *p* = 0.001).

Image parameters showed that the ET diameter and MFL diameter in the non-surviving patients were relatively more extensive than in the surviving group (8.1 vs. 5 mm, *p* = 0.003; 27.4 vs. 24.1 mm, *p* = 0.096, respectively). There was no significant difference in other image variables for type A AAD.

### Logistic Regression Analysis and Final Prognostic Model Derivation and Validation in AAD Patients

Multivariate logistic regression analysis was performed to predict the in-hospital mortality in AAD patients. It showed that predictors of in-hospital mortality included age [odds ratio (OR), 1.051; 95% confidence interval (CI), 1.009–1.094; *p* = 0.017], type A AAD (OR, 22.354; CI, 4.665–107.107; *p* < 0.001), Marfan syndrome (OR, 7.223; CI, 1.185–44.033; *p* = 0.032), pericardial effusion (OR, 3.423; CI, 1.124–10.428; *p* = 0.030), and MFL diameter (OR, 1.049; CI, 1.009–1.092; *p* = 0.017). Surgical repair was protective against in-hospital death (OR, 0.231; CI, 0.090–0.594; *p* = 0.002; Table 2).

**Table 2:** Multivariate logistic analysis of potential prognostic factors in AAD patients.

**Factor**	**Multivariable OR (95% CI)**	***P*-Value**
Age	1.051 (1.009, 1.094)	**0.017**
Stanford A	22.354 (4.665, 107.107)	**<0.001**
MFS	7.223 (1.185, 44.033)	**0.032**
Surgical repair	0.231 (0.090, 0.594)	**0.002**
Pericardial effusion	3.423 (1.124, 10.428)	**0.030**
Site of intimal tear		1.151
0 (none)		
1 (ascending aorta)		
2 (aortic arch)		
3 (thoracoabdominal aorta)		
Entery tear diameter	1.017 (0.939, 1.103)	0.676
Maximum false lumen diameter	1.049 (1.009, 1.092)	**0.017**

*Bold values are used to indicate the significance of these indexes in multivariate analysis (P < 0.05)*.

**Figure 1:**
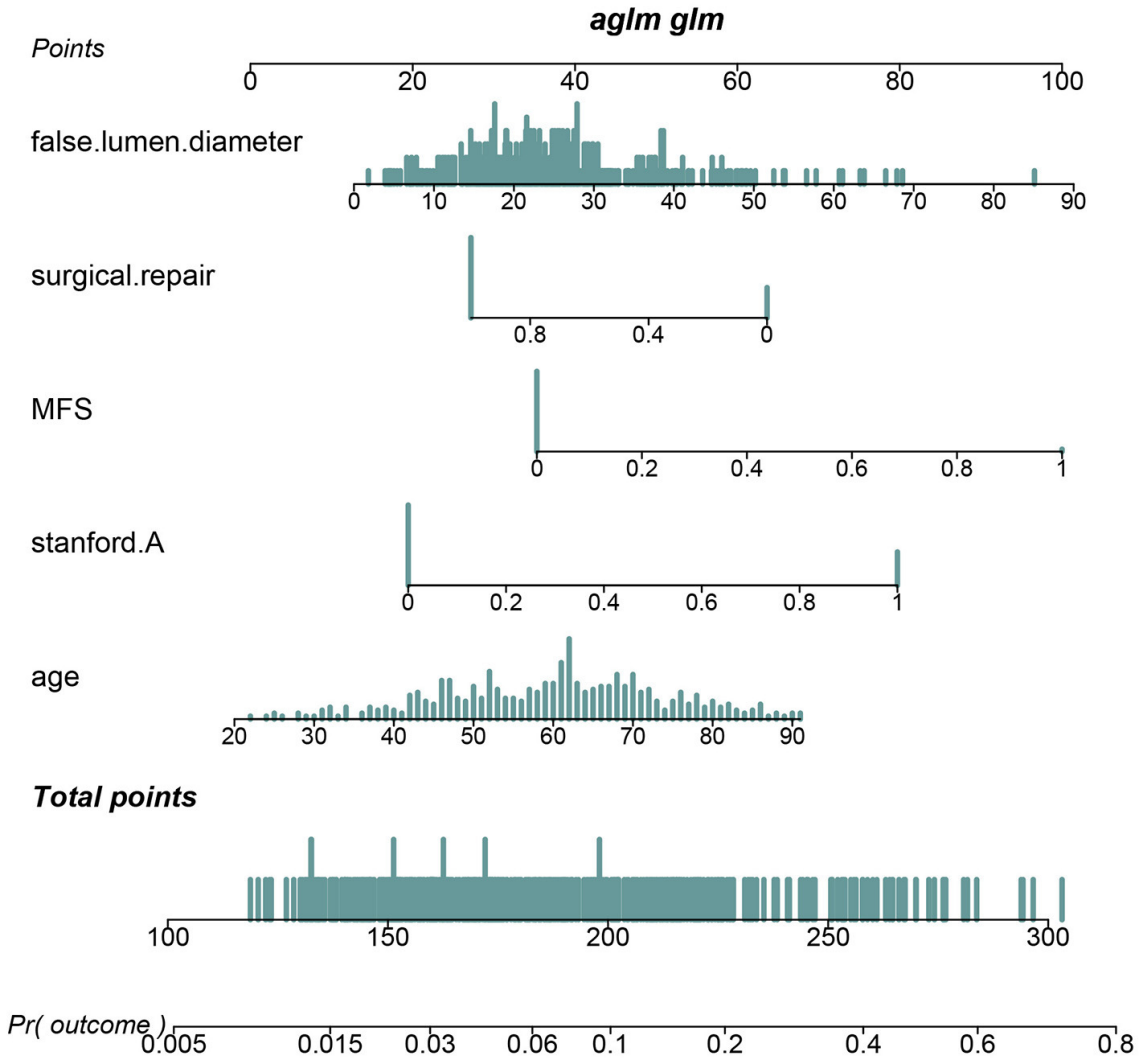
A nomogram for predicting the in-hospital prognosis of patients with AAD.

**Figure 2:**
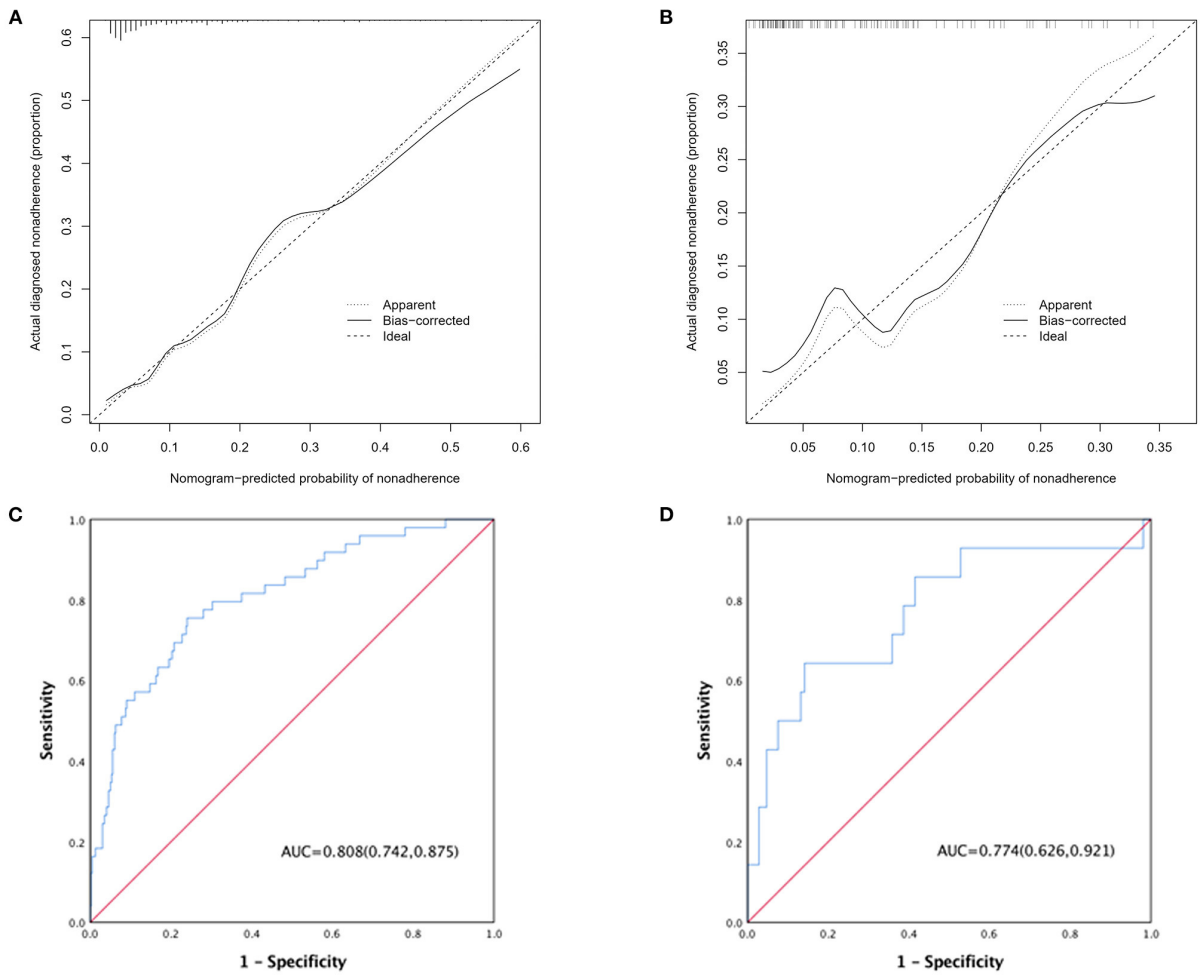
**(A)** Calibration curve of the AAD nomogram in the derivation cohort, which depicts the calibration of the nomogram in terms of the agreement between the predicted risk of death and observed outcomes. The 45° dotted line represents an ideal prediction, and the solid line represents the bias-corrected predictive performance of the nomogram. The closer the solid line fits to the ideal line, the better the predictive accuracy of the nomogram; **(B)** calibration curve in the validation cohort; **(C)** ROC curve of the AAD nomogram in the derivation cohort; **(D)** ROC curve in the validation cohort.

Pericardial perfusion was removed from consideration because it failed to offer a significant improvement in model fit, as suggested by the likelihood ratio test (Supplementary Table 3). Hence, the final prognostic model based on five variables, namely, age, type A AAD, Marfan syndrome, surgical repair, and MFL diameter, was constructed (Figure 1). The C-index value was 0.808 (0.742–0.875) in the derivation cohort and 0.774 (0.626–0.921) in the validation cohort. The calibration curves of the final prognostic model showed high consistencies between the predicted and observed survival probability in both the derivation (Figure 2A) and validation cohorts (Figure 2B). The AUCs of the final prognostic model in the derivation and validation cohorts were 0.808 (0.742–0.875; Figure 2C) and 0.774 (0.626–0.921; Figure 2D), respectively, which implied successful discrimination. The model achieved an AUC of 0.7918 and an *R* square of 0.1515 in the 10-fold cross-validation.

### Logistic Regression Analysis and 3ADPS Derivation and Validation in Type A AAD Patients

Multivariate logistic regression analysis of type A AAD patients showed that predictors of in-hospital mortality included Marfan syndrome (OR, 17.810; CI, 2.021–97.390; *p* = 0.01), the site of intimal tear (*p* = 0.001), pericardial effusion (OR, 3.431; CI, 1.008–11.675; *p* = 0.049), and MFL diameter (OR, 1.069; CI, 1.016–1.125; *p* = 0.01). Similarly, surgical repair was protective against in-hospital death (OR, 0.075; CI, 0.021–0.269; *p* < 0.001; Supplementary Table 4). The above 5 variables were included in the final model (3-level type A aortic dissection clinical prognosis score, 3ADPS), and we assigned points for each of them according to the regression coefficient. The final model was presented in Table 3.

**Table 3:** Three-level type A aortic dissection clinical prognosis score (3ADPS).

**Variable**	**Regression coefficient**		**Points**
MFS	2.211	No	0
		Yes	2
Surgical repair	−2.197	No	0
		Yes	−2
Pericardial effusion	0.821	No	0
		Yes	1
Maximum false lumen diameter <22 mm	Reference	<22 mm	0
22 mm ≤ False lumen diameter <45 mm	1.704	22–45 mm	1
45 mm ≤ False lumen diameter	3.721	≥45 mm	3
Intimal tear in the aortic arch or descending aorta	2.689	No	0
		Yes	2
In-hospital death risk, total			
Moderate risk of death (<20%)	<0		
High risk of death (20–50%)	0–2		
Very high risk of death (>50%)	≥3		

**Figure 3:**
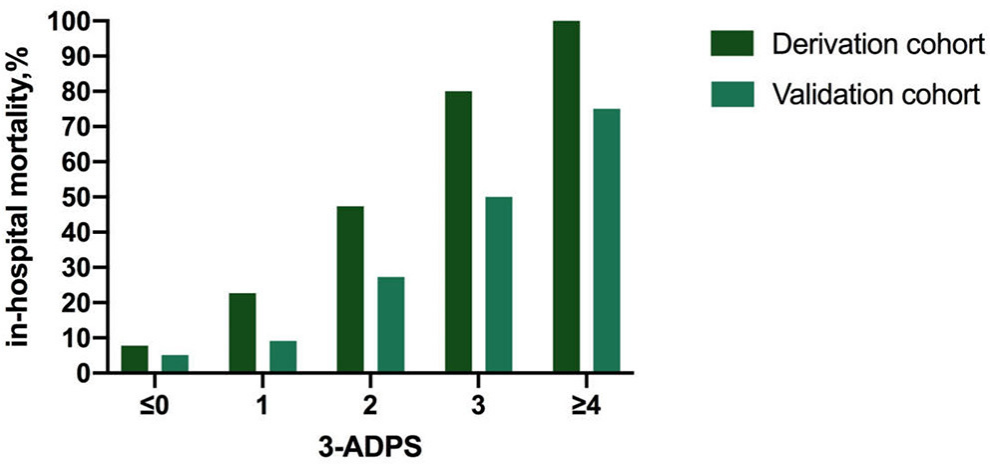
The in-hospital outcome of type A AAD was evaluated by 3ADPS and the distribution of 3ADPS in the derivation and validation cohorts.

**Figure 4:**
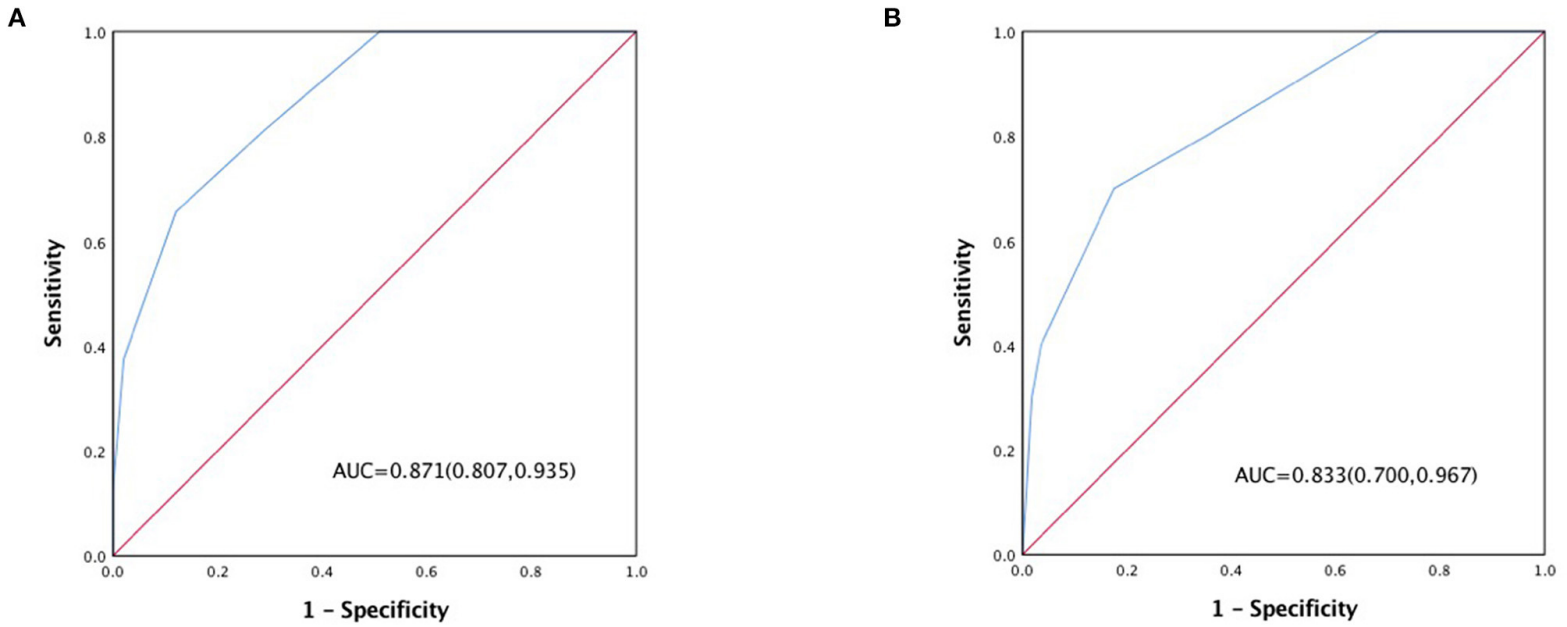
**(A)** ROC curve of the 3ADPS in the derivation cohort and **(B)** ROC curve in the validation cohort.

The in-hospital outcome of type A AAD evaluated by 3ADPS and the distribution of 3ADPS in the derivation cohort were presented in Figure 3. According to the predefined cutoff values, a 3ADPS < 0 represents a moderate risk of death (<20%), a 3ADPS of 0–2 represents a high risk of death (<50%), a 3ADPS more than 3 represents a very high risk of death (50% or greater) (Table 3). In the derivation cohort, the AUC was 0.871 (0.807–0.935) (Figure 4A).

For the validation cohort, the in-hospital outcome of type A AAD evaluated by 3ADPS and the distribution of 3ADPS in the derivation cohort are presented in Figure 3. In the validation cohort, the AUC was 0.833 (0.700–0.967) (Figure 4B).

## Discussion

It is reported that untreated patients with AAD have a significant mortality rate of 1–2% per hour immediately after symptom onset, primarily type A AAD (3, 5). Previous studies showed that in-hospital mortality for type A AAD was up to 26% for open surgical procedures and 58% for medical management (6, 22). Therefore, it is necessary but full of challenges for emergency doctors to determine the in-hospital prognosis of AAD patients for successful management. This study aimed to construct bedside risk prediction tools mainly based on image parameters to determine the in-hospital outcomes of AAD in ED.

This study identified that age, type A AAD, Marfan syndrome, surgical repair, pericardial effusion, and MFL diameter were independent predictors of mortality in AAD patients. The clinical symptoms and signs of elderly patients are often atypical, leading to delayed diagnosis and a bad prognosis. IRAD reported that patients aged ≥ 70 years had an overall higher in-hospital mortality than patients aged < 70 years (43 vs. 28% for type A AAD, 16 vs. 10% for type B AAD, *p* < 0.05) (5, 17, 23), which was consistent with our results. It is well-known that Marfan syndrome is the most common heritable connective tissue aortic disorder and causes aortic root enlargement in 60–80% of patients (11, 24). Therefore, type A was more frequent than type B in patients with Marfan syndrome, and the majority of patients with type A AAD and Marfan syndrome with dissection involving only the ascending aorta or more segment and aortic rupture had higher in-hospital mortality if not surgically repaired in time. This study confirmed it in the derivation cohort (mortality rate in non-surviving patients 10.2 vs. 2% in surviving patients, *p* < 0.001, Supplementary Table 1).

There is a consensus that AAD is an urgent surgical emergency, primarily type A AAD, and IRAD data have further confirmed that patients treated medically alone had remarkably higher in-hospital mortality than those who received surgical repair simultaneously (58.1 vs. 23.9%). Since the late 1990s, most patients with type A AAD have been managed surgically, rising from 79 to 90% (5, 8). More operative procedures, including a valve-sparing root repair, an ascending with hemi or complete arch replacement, and frozen elephant trunk deployment if needed, were implemented in recent years. And in-hospital mortality rate of type A AAD decreased significantly from 31 to 22% over time, mainly because of decreased surgical mortality (8, 15). Endovascular management tends to be the first-line therapy for type B AAD patients complicated by malperfusion syndrome, progression of dissection, rapid aortic expansion, or instability hemodynamic, while medical management was still reserved for patients who had an uncomplicated course (14, 25). This study implied that more AAD patients in the surviving group received surgical repair than the non-surviving group (75.8 vs. 46.9%, *p* < 0.001), and more type A AAD patients in the surviving group received open surgery compared with the non-surviving group (71 vs. 37.5%, *p* = 0.001), which were following previous reports.

The presence of pericardial effusion indicates the destruction of the integrity of the outer aortic wall. Patients with pericardial effusion are more likely to have a periaortic hematoma, and pericardial tamponade may occur when pericardial effusion suddenly increases. The mortality of patients with pericardial tamponade remained dramatically high, and periaortic hematomas were identified to be an independent predictor for AAD (26, 27). CTA can easily identify the presence of pericardial effusion, and echocardiography can also be performed. These image findings provided important diagnostic information, had prognostic value, and were helpful to optimize treatment. Bossone, Eduardo et al. found that evidences of pericardial effusion, pericardial tamponade, and periaortic hematoma were more frequent in non-survivors (51.1 vs. 40.9%, *p* = 0.04; 34.5 vs. 11.3%, *p* < 0.001; 23.8 vs. 14.7%, *p* = 0.02, respectively), (28). As expected, both the univariate and multivariate regression analysis of this study suggested that pericardial effusion was a satisfactory predictor of mortality in patients with AAD [3.600 (1.787, 7.254), *p* < 0.001; 3.423 (1.124, 10.428), *p* = 0.03; respectively]. Thus, the presence of pericardial effusion indicates a poor prognosis and should warrant urgent surgical intervention.

Several image variables such as initial false lumen diameter and patent or partially false lumen thrombosis have been proved to be high-risk features indicating unstable disease (including aneurysmal growth and need for late intervention) in apparently stable type B AAD patients. In addition, a larger false lumen implied poorer organ perfusion and was confirmed to be associated with an unsatisfactory long-term survival in type B AAD (14, 29). In contrast, few studies have focused on the relationship between false lumen diameter and in-hospital prognosis in type A AAD. This study showed that larger maximum false lumen (MFL) diameter was more frequent in non-survivors both in AAD and type A AAD derivation cohorts (27.5 vs. 24.5 mm, *p* = 0.019; 27.4 vs. 24.1 mm, *p* = 0.096, respectively). Accordingly, multivariate regression analysis also implied MFL is a good predictor of mortality both in AAD and type A AAD derivation cohorts (OR, 1.049, *p* = 0.017; OR, 1.06927.4, *p* = 0.01, respectively). In addition, patients with an intimal tear originating in the aortic arch or thoracoabdominal aorta were confirmed to suffer a poor in-hospital outcome in the type A AAD derivation cohort, which was due to the greater extent of dissection and the independent entity from the perspective of the pathology undoubtedly. Also, the site of the intimal tear (aortic arch and thoracoabdominal aorta) was a perfect predictor of mortality in the type A AAD derivation cohort by multivariate regression analysis (OR, 71.738, *p* = 0.001; OR, 136.125, *p* < 0.001, respectively).

Once the patient with AAD presents to the emergency room, simple bedside tools for estimating in-hospital outcomes were helpful for emergency doctors to make a rapid and correct prognosis prediction and ensure management without any delay. Meanwhile, family members could also be fully informed of the patient's risk conditions and consider available treatment. The final prognostic model of AAD incorporating age, Marfan syndrome, type A AAD, surgical repair, and MFL diameter, showed good calibration and discrimination in the derivation and validation cohorts. As for type A AAD, 3ADPS was confirmed acceptable calibration and accuracy. As multivariable risk prediction tools, the models were readily available for emergency doctors to predict in-hospital mortality of AAD patients in extreme clinical risk.

Nevertheless, this study has some limitations. It is a single-center study and lacks external validation cohorts. The population for each cohort is relatively small. And there was a bias in the number of type A and B AAD between the two cohorts, which was due to more patients with type A AAD transferred to our hospital because of the multidisciplinary expertise and excellent infrastructure. The nomogram and 3ADPS strategy need to be formally validated in a more enormous prospective, multicenter implementation study.

## Conclusions

The age, Marfan syndrome, type A AAD, surgical repair, and MFL diameter can significantly affect the in-hospital outcomes of AAD. And 3ADPS contributes to the prediction of in-hospital prognosis of type A AAD rapidly and effectively. The simple bedside tools for estimating in-hospital outcomes were helpful for emergency doctors to make a rapid and correct prognosis prediction for AAD patients in extreme clinical risk and then ensure management without any delay.

## Data Availability Statement

The original contributions presented in the study are included in the article/Supplementary Materials, further inquiries can be directed to the corresponding author/s.

## Ethics Statement

The study was approved by the Ethics Committee of Zhongshan Hospital Fudan University (Shanghai, China) (Record Number B2019-319R). The patients/participants provided their written informed consent to participate in this study.

## Author Contributions

GG and CY conceived, designed, and coordinated the study. LX and YH drafted this manuscript. MT, ZD, XZ, and SG were responsible for collecting and measuring the imaging data. DC, YY, and FL were responsible for collecting clinical data. YZ and CC were responsible for data analysis. All authors read, approved, and contributed to the final manuscript.

## Funding

This study was supported by the Clinical Research Project of Zhongshan Hospital (2020ZSLC46 and 2020ZHZS33), Clinical Research Project of Shanghai Science and Technology Committee (21Y11902200), and Clinical Research Project of Shanghai Municipal Health Commission (201940163).

## Conflict of Interest

ZD was employed by Suzhou Zhi Zhun Medical Technology Co., Ltd., and SG was employed by CANON Medical Systems (China) Co., Ltd. Shanghai Huidr Information Technology Co., Ltd assisted in following up with the patients. The remaining authors declare that the research was conducted in the absence of any commercial or financial relationships that could be construed as a potential conflict of interest.

## Publisher's Note

All claims expressed in this article are solely those of the authors and do not necessarily represent those of their affiliated organizations, or those of the publisher, the editors and the reviewers. Any product that may be evaluated in this article, or claim that may be made by its manufacturer, is not guaranteed or endorsed by the publisher.

## Supplementary Material

Supplementary Figure 1 A flowchart of this study.

Supplementary Figure 2 **(A)** On the oblique coronary section, line-a is a line drawn parallel to the curve aortic ascendens or arch, and line-b is a line perpendicular to line-a. The red arrow shows the direction of blood flow in the true lumen, and the dark red arrow shows the direction of blood flow in the false lumen; **(B)** On cross-section, line-a is a line drawn parallel to the curve aortic ascendens or arch, and line-b is a line perpendicular to the line-a. A reproducible manual method was used to measure the maximal diameter of FL and the minimal diameter of TL on line-b.

Supplementary Table 1 Comparison between surviving and non-surviving AAD patients in the derivation cohort.

Supplementary Table 2 Comparison between surviving and non-surviving type A AAD patients in the derivation cohort.

Supplementary Table 3 Likelihood ratio test of different prediction models regarding in-hospital outcome of AAD in the training cohort. AAD, acute aortic dissection; MFS, Marfan syndrome; MFL, maximum false lumen; AIC, Akaike Information Criterion.

Supplementary Table 4 Multivariate logistic analysis of potential prognostic factors in type A AAD patients.

## Abbreviations

AAD: acute aortic dissection
CTA: computed tomography angiography
ET: entry tear
MFL: maximum false lumen diameter
3ADPS: 3-Level type A aortic dissection clinical prognosis score
IMH: intramural hematoma
PAU: penetrating aortic ulcer
ED: emergency department
AAS: acute aortic syndrome
CTPA: computed tomography pulmonary angiography
C-index: concordance index
AUC: area under the receiver operating characteristic curve.
