# Supplementary figures and images for: Simple Death Risk Models to Predict In-hospital Outcomes in Acute Aortic Dissection in Emergency Department
Source: Front Med (Lausanne). 2022 May 23;9:890567. doi: 10.3389/fmed.2022.890567 (PMC9168913; doi:10.3389/fmed.2022.890567)

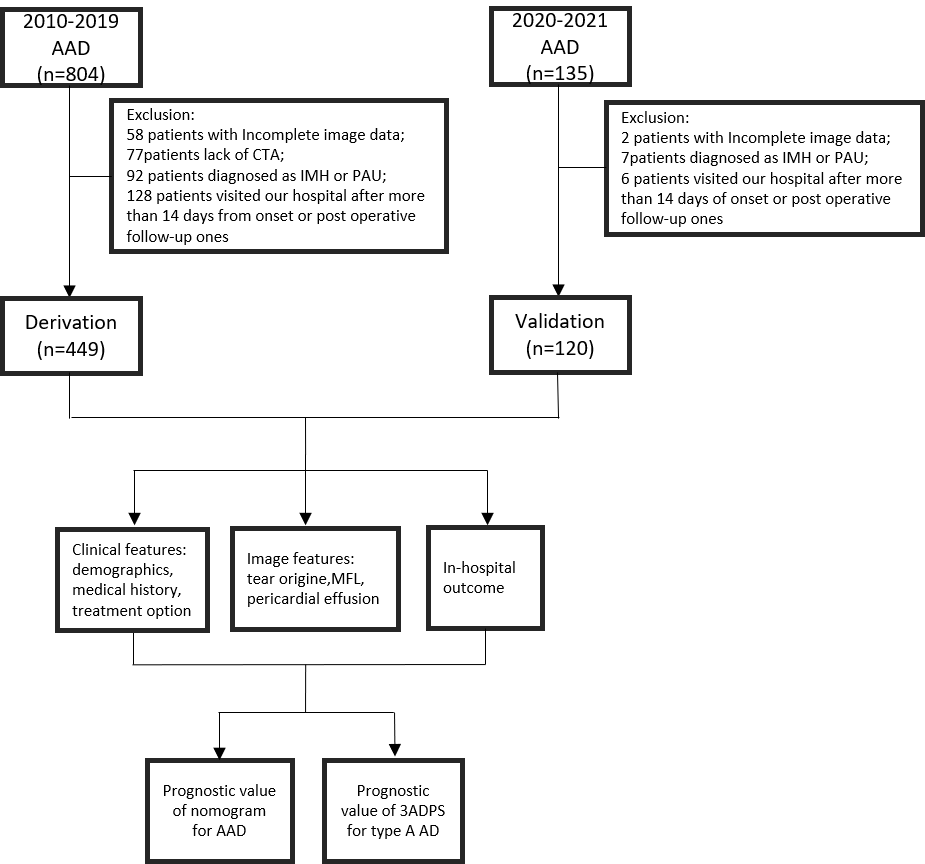

Supplement: Supplementary Figure 1 — A flowchart of this study. [file Image_1.tif]

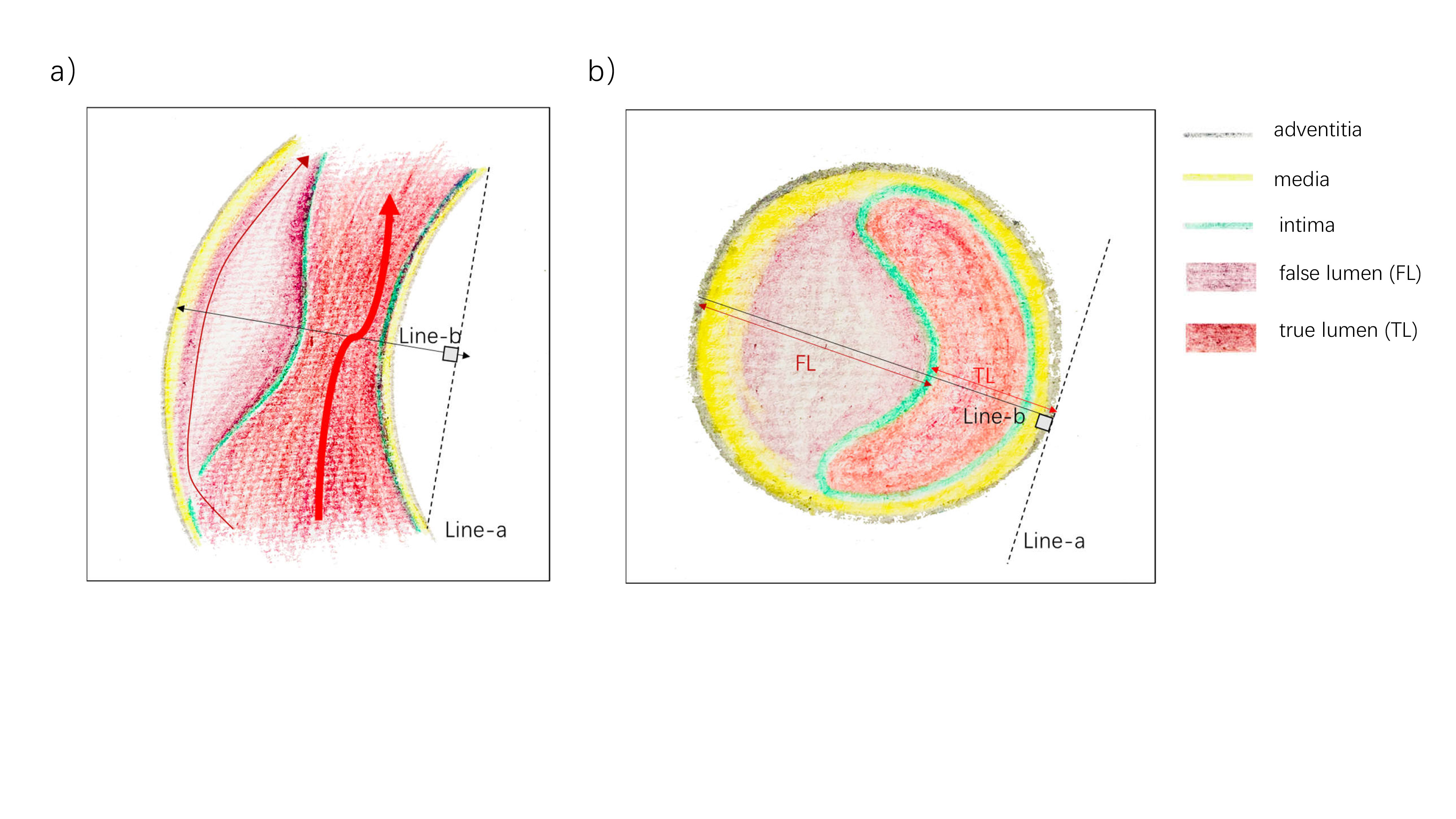

Supplement: Supplementary Figure 2 — (A) On the oblique coronary section, line-a is a line drawn parallel to the curve aortic ascendens or arch, and line-b is a line perpendicular to line-a. The red arrow shows the direction of blood flow in the true lumen, and the dark red arrow shows the direction of blood flow in the false lumen; (B) On cross-section, line-a is a line drawn parallel to the curve aortic ascendens or arch, and line-b is a line perpendicular to the line-a. A reproducible manual method was used to measure the maximal diameter of FL and the minimal diameter of TL on line-b. [file Image_2.jpeg]
