# Supplementary material for: Simple Death Risk Models to Predict In-hospital Outcomes in Acute Aortic Dissection in Emergency Department
Source: Front Med (Lausanne). 2022 May 23;9:890567. doi: 10.3389/fmed.2022.890567 (PMC9168913; doi:10.3389/fmed.2022.890567)
Supplement: Supplementary Table 1 — Comparison between surviving and non-surviving AAD patients in the derivation cohort. [file Data_Sheet_1.docx]

**Supplementary Table 1. Comparison between surviving and non-surviving AAD patients in the derivation cohort**

|  | Surviving group  (n=400) | Non-surviving group  (n=49) | P value |
| --- | --- | --- | --- |
| Age | 61(49,69) | 62(53,70) | 0.275 |
| Sex, male | 307(76.8%) | 38(77.6%) | 0.900 |
| AAD type |  |  | <0.001 |
| Stanford A | 99(24.8%) | 33(67.3%) |  |
| Stanford B | 301(75.3%) | 16(32.7%) |  |
| History |  |  |  |
| hypertension | 271(67.8%) | 27(55.1%) | 0.077 |
| Diabete | 32(8%) | 3(6.1%) | 0.857 |
| MFS | 8(2%) | 5(10.2%) | 0.001 |
| AA | 51(12.8%) | 11(22.4%) | 0.063 |
| Surgical history of AA/AD | 17(4.3%) | 4(8.2%) | 0.386 |
| CAD/HF | 40(10%) | 7(14.3%) | 0.355 |
| Surgical repair | 303(75.8%) | 23(46.9%) | <0.001 |
| Endovascular therapy | 227(56.8%) | 10(20.4%) | <0.001 |
| Open surgery | 77(19.3%) | 14(28.6%) | 0.126 |
| Surgery within 14 days | 227(56.8%) | 22(44.9%) | 0.115 |
| Time from onset to surgery | 8(5,14) | 4(1,6) | <0.001 |
| Hospital stay (days) | 18±11 | 32±47 | 0.259 |
| Imaging parameter |  |  |  |
| Ascending aorta - aortic arch | 129(32.3%) | 30(61.2%) | <0.001 |
| Aortic arch - celiac trunk | 366(91.5%) | 39(79.6%) | 0.008 |
| Celiac trunk - lliac artery | 323(80.8%) | 37(75.5%) | 0.385 |
| Extent of dissection |  |  |  |
| 0(arotic arch - thoracoabdominal aorta) | 272(68%) | 19(38.8%) | <0.001 |
| 1(ascending aorta - aortic arch) | 20(5%) | 8(16.3%) | 0.002 |
| 2(ascending aorta - thoracoabdominal aorta) | 109(27.3%) | 22(44.9%) | 0.010 |
| Involved vessels |  |  |  |
| Aortic sinus | 12(3%) | 7(14.3%) | <0.001 |
| Left coronary artery | 0(0%) | 0(0%) | - |
| Right coronary artery | 0(0%) | 0(0%) | - |
| Brachiocephalic trunk | 32(8%) | 14(28.6%) | <0.001 |
| Left common carotid artery | 30(7.5%) | 12(24.5%) | <0.001 |
| Left subclavian artery | 34(8.5%) | 11(22.4%) | 0.002 |
| Celiac trunk | 21(5.3%) | 6(12.2%) | 0.052 |
| Superior mesenteric artery | 26(6.5%) | 7(14.3%) | 0.049 |
| Inferior mesenteric artery | 0(0%) | 0(0%) | - |
| Renal artery | 20(5%) | 4(8.2%) | 1.000 |
| Common iliac artery | 121(30.3%) | 21(42.9%) | 0.073 |
| Aortic diameter |  |  |  |
| Aortic root | 27.1(24.8,29.8) | 26.2(24.4,29.7) | 0.364 |
| Aortic sinus | 36.6(33.2,40.9) | 39.7(33.8,44.1) | 0.06 |
| Left subclavian artery level | 29.9±4.8 | 31.2±7.2 | 0.027 |
| Left renal artery level | 22.3±5.5 | 24.7±7.1 | 0.081 |
| Superior mesenteric artery level | 24.3±6.2 | 25.5±6.3 | 0.185 |
| Inferior mesenteric artery level | 22.6±8.1 | 21.9±5.8 | 0.591 |
| Minimum true lumen diameter | 7.8(5.1,11) | 9.3(4.8,11.9) | 0.530 |
| Maximum false lumen diameter | 24.5±10.6 | 27.5±15.4 | 0.019 |
| Entry tear diameter | 3.9(0,8) | 7.1(3.4,12.6) | 0.001 |
| Tear condition |  |  |  |
| Anterograde tear | 243(60.8%) | 37(75.5%) | 0.044 |
| Sleeve tear | 90(22.5%) | 7(14.3%) | 0.187 |
| Site of intimal tear |  |  |  |
| 0 (none) | 160(40%) | 11(22.4%) | 0.017 |
| 1 (ascending aorta) | 50(12.5%) | 17(34.7%) | <0.001 |
| 2 (aortic arch) | 28(7%) | 8(16.3%) | 0.023 |
| 3 (thoracoabdominal aorta) | 162(40.5%) | 13(26.5%) | 0.058 |
| Other imaging parameter |  |  |  |
| Aortic exudation | 9(2.3%) | 2(4.1%) | 0.769 |
| Pericardial effusion | 40(10%) | 14(28.6%) | <0.001 |
| Pleural effusion | 114(28.5%) | 18(36.7%) | 0.232 |

**Supplementary Table 2. Comparison between surviving and non-surviving type A AAD patients in the derivation cohort**

|  | Surviving group  (n=100) | Non-surviving group  (n=32) | P value |
| --- | --- | --- | --- |
| Age | 57(46,67) | 59(51,64) | 0.608 |
| Sex, male | 71(73%) | 26(81.3%) | 0.358 |
| History |  |  |  |
| hypertension | 56(56%) | 17(53.1%) | 0.839 |
| Diabete | 7(7%) | 1(3.1%) | 0.679 |
| MFS | 4(4%) | 4(12.5%) | 0.097 |
| AA | 10(10%) | 5(15.6%) | 0.358 |
| Surgical history of AA/AD | 5(5%) | 1(3.1%) | 1.000 |
| CAD/HF | 16(16%) | 3(9.4%) | 0.563 |
| Surgical repair | 79(79%) | 12(37.5%) | <0.001 |
| Endovascular therapy | 8(8%) | 1(3.1%) | 0.687 |
| Open surgery | 71(71%) | 12(37.5%) | 0.001 |
| Surgery within 14 days | 60(60%) | 12(37.5%) | 0.040 |
| Time from onset to surgery | 5(1,13) | 2(1,5) | 0.124 |
| Hospital stay (days) | 24±14 | 56±77 | 0.396 |
| Imaging parameter |  |  |  |
| Ascending aorta - aortic arch | 88(88%) | 26(81.3%) | 0.377 |
| Aortic arch - celiac trunk | 81(81%) | 25(78.1%) | 0.799 |
| Celiac trunk - lliac artery | 69(69%) | 24(75%) | 0.657 |
| Extent of dissection |  |  |  |
| 0(arotic arch - thoracoabdominal aorta) | 12(12%) | 6(18.8%) | 0.377 |
| 1(ascending aorta - aortic arch) | 16(16%) | 7(21.9%) | 0.434 |
| 2(ascending aorta - thoracoabdominal aorta) | 72(72%) | 19(59.4%) | 0.194 |
| Involved vessels |  |  |  |
| Aortic sinus | 12(12%) | 6(18.8%) | 0.377 |
| Left coronary artery | - | - |  |
| Right coronary artery | - | - |  |
| Brachiocephalic trunk | 29(29%) | 14(43.8%) | 0.134 |
| Left common carotid artery | 25(25%) | 11(34.4%) | 0.362 |
| Left subclavian artery | 25(25%) | 10(31.3%) | 0.497 |
| Celiac trunk | 13(13%) | 5(15.6%) | 0.769 |
| Superior mesenteric artery | 13(13%) | 6(18.8%) | 0.401 |
| Inferior mesenteric artery | - | - |  |
| Renal artery | 10(10%) | 4(12.5%) | 0.743 |
| Common iliac artery | 31(31%) | 13(40.6%) | 0.389 |
| Aortic diameter |  |  |  |
| Aortic root | 27.1(24.5,30.4) | 25.5(24.3,30) | 0.388 |
| Aortic sinus | 40.5(35.9,46) | 40.3(33.8,44.5) | 0.394 |
| Left subclavian artery level | 30.6±4.3 | 32±7.3 | 0.085 |
| Left renal artery level | 22.2(19.5,24.6) | 22.6(20.2,27.3) | 0.259 |
| Superior mesenteric artery level | 23.2(20.5,25.3) | 24.4(21.5,27.4) | 0.142 |
| Inferior mesenteric artery level | 19.7(17.1,22.9) | 19.6(18.3,24.4) | 0.537 |
| Minimum true lumen diameter | 6.6(4.1,10) | 9(4.3,12) | 0.301 |
| Maximum false lumen diameter | 24.1(20.1,34.3) | 27.4(24,34.4) | 0.096 |
| Entry tear diameter | 5(0,9) | 8.1(4.6,12.9) | 0.003 |
| Tear condition |  |  |  |
| Anterograde tear | 66(66%) | 29(90.6%) | 0.006 |
| Sleeve tear | 21(21%) | 2(6.3%) | 0.064 |
| Site of intimal tear |  |  |  |
| 0 (none) | 34(34%) | 2(6.3%) | 0.001 |
| 1 (ascending aorta) | 47(47%) | 16(50%) | 0.840 |
| 2 (aortic arch) | 7(7%) | 6(18.8%) | 0.082 |
| 3 (thoracoabdominal aorta) | 12(12%) | 8(25%) | 0.091 |
| Other imaging parameter |  |  |  |
| Aortic exudation | 1(1%) | 1(3.1%) | 0.427 |
| Pericardial effusion | 22(22%) | 13(40.6%) | 0.064 |
| Pleural effusion | 30(30%) | 14(43.8%) | 0.196 |

| **Supplementary Table 3. Likelihood ratio test of different prediction models regarding in-hospital outcome of AAD in the training cohort.** | | | | | |
| --- | --- | --- | --- | --- | --- |
| Models | Variables included in the model | AIC | Log likelihood | Chi square | P |
| 1 (Full Model) | Age+Stanford A+MFS+Surgical repair+Maximum false lumen diameter+Pericardial effusion | 258.44 | -122.22 | - | - |
| 2 (Without Age) | Stanford A+MFS+Surgical repair+Maximum false lumen diameter+Pericardial effusion | 261.45 | -124.72 | 5.0121 | 0.025 |
| 3 (Without Stanford A) | Age+MFS+Surgical repair+Maximum false lumen diameter+Pericardial effusion | 279.73 | -133.87 | 23.395 | <0.001 |
| 4 (Without MFS) | Age+Stanford A+Surgical repair+Maximum false lumen diameter+Pericardial effusion | 264.25 | -126.12 | 7.8099 | 0.005 |
| 5 (Without Surgical repair) | Age+Stanford A+MFS+Maximum false lumen diameter+Pericardial effusion | 266.07 | -127.04 | 9.6369 | 0.002 |
| 6 (Without MFL diameter) | Age+Stanford A+MFS+Surgical repair+Pericardial effusion | 261.16 | -124.58 | 4.7187 | 0.030 |
| 7 (Without Pericardial effusion) | Age+Stanford A+MFS+Surgical repair+Maximum false lumen diameter | 257.81 | -122.91 | 1.3774 | 0.2405 |

AAD, acute aortic dissection; MFS, Marfan syndrome; MFL, maximum false lumen; AIC, Akaike Information Criterion

**Supplementary Table 4. Multivariate logistic analysis of potential prognostic** **factors in type A AAD patients**

| Factor | Multivariable OR  (95% CI) | P value |
| --- | --- | --- |
| Age | 1.033（0.974，1.096） | 0.280 |
| Male | 3.790（0.857，16.748） | 0.079 |
| MFS | 17.810（2.021，97.390） | **0.010** |
| Surgical repair | 0.075（0.021，0.269） | **<0.001** |
| pericardial effusion | 3.431（1.008，11.675） | **0.049** |
| Site of intimal tear |  | **0.001** |
| 0 (none) | Reference |  |
| 1 (ascending aorta) | 12.670（1.481，108.399） | 0.020 |
| 2 (aortic arch) | 71.738（5.922，869.072） | 0.001 |
| 3 (thoracoabdominal aorta) | 136.125（9.994，1854.049） | <0.001 |
| Entry tear diameter | 0.974（0.887，1.070） | 0.583 |
| Maximum false lumen diameter | 1.069（1.016，1.125） | **0.010** |
